# Mortality rates and division of labor in the leaf-cutting ant, Atta colombica

**DOI:** 10.1673/2006_06_18.1

**Published:** 2006-09-19

**Authors:** Mark J F. Brown, A N M. Bot, Adam G. Hart

**Affiliations:** 1 Ecology and Evolution, ETH-Zürich, Switzerland; 2 Department of Zoology, School of Natural Sciences, Trinity College Dublin, Dublin 2, Ireland; 3 Department of Genetics and Ecology, University of Århus, Denmark; 4 (current address) Section of Evolutionary Biology, Institute of Evolutionary and Ecological Sciences, University of Leiden, Kaiserstraat 63, 2311 GP Leiden, The Netherlands; 5 Smithsonian Tropical Research Institute, Panama City, Panama; 6 Department of Animal and Plant Sciences, University of Sheffield; 7 Department of Natural & Social Sciences, School of Environment, University of Gloucestershire, Francis Close Hall, Swindon Road, Cheltenham, Gloucestershire, GL50 4AZ

**Keywords:** social insects, task allocation, waste management, hygiene

## Abstract

Division of labor in social groups is affected by the relative costs and benefits of conducting different tasks. However, most studies have examined the dynamics of division of labor, rather than the costs and benefits that presumably underlie the evolution of such systems. In social insects, division of labor may be simplistically described as a source-sink system, with external tasks, such as foraging, acting as sinks for the work force. The implications of two distinct sinks – foraging and waste-heap working – for division of labor were examined in the leaf-cutting ant Atta colombica. Intrinsic mortality rates were similar across external task groups. Exposure to waste (a task-related environment) led to a 60% increase in the mortality rate of waste-heap workers compared to workers not exposed to waste. Given the small number of workers present in the waste-heap task group, such increases in mortality are unlikely to affect division of labor and task allocation dramatically, except perhaps under conditions of stress.

## Introduction

Division of labor plays a central role in the organisation and success of social groups (Oster and Wilson 1978). By enabling groups to coordinate their response to challenges (e.g.,Gordon 1986), division of labor is assumed to promote ergonomic efficiency (Oster and Wilson 1978) and provide ecological benefits (e.g., predator evasion; McGowan and Woolfenden 1989) from which all group members can benefit. Consequently, an understanding of the behavioural rules and causal factors underlying division of labor will enhance our overall understanding of the evolution of social groups. Analyses of behavioural rules and causal factors have tended to employ a cost/benefit approach (pioneered by Oster and Wilson 1978), where the benefits of a particular aspect of division of labor are compared to its costs at the individual level, the group level, or both (e.g., Bednekoff 1997). However, there is little hard evidence for the underlying assumption that costs and benefits are crucial to the structuring of division of labor (Schmid-Hempel 1992; but see Clutton-Brock et al. 1998, 1999).

Social insects have complex and well-studied systems of division of labor (Wilson 1975; Gordon 1996; Beshers and Fewell 2001), and studies of insect societies have generated both ultimate and proximate explanations for how division of labor is structured (Beshers and Fewell 2001). Proximate explanations have included genetic variation (Fuchs and Moritz 1998), behavioural thresholds (Beshers et al. 1999), interaction patterns (Gordon 1999), age (Wilson 1976), physiology (Powell and Tschinkel 1999; Blanchard et al. 2000), and source-sink models of worker movement (Tofts 1993). Assuming that division of labor and worker allocation are adaptive, their structure should be the product of the associated costs and benefits of particular strategies. That costs are important is suggested by observations such as high levels of worker inactivity (Cole 1986), changes in foraging in response to overabundant food (Rissing 1989) or mortality pressure (Gentry 1974) and general flexibility in worker allocation to different tasks as the environment changes (Gordon 1986,^1987^, ^1989^, ^1991^; Calabi and Traniello 1989; Crosland and Traniello 1997). However, the costs and benefits associated with different tasks, or patterns of task allocation and division of labor, have rarely been measured and may not always be tractable [but see for foraging costs De Vita (1979),Porter and Jorgensen (1981), Schmid-Hempel and Schmid-Hempel (1984), Weier and Feener (1995), Fewell et al. (1996) and for trail maintenance costs Howard (2001)].

Insect societies normally have a workforce functionally split into an internal work group (or innendienst), tending the brood and performing other intra-nidal duties, and an external work group (aussendienst) gathering forage and defending the nest or territory (Hölldobler and Wilson 1990). The usual pattern of temporal polyethism is that internal workers become external workers towards the end of their lives (Hölldobler and Wilson 1990). However, an asymmetry in mortality rates between external and internal workers (with external workers generally facing higher mortality rates than internal workers; Schmid-Hempel and Schmid-Hempel 1984) can result in a pull of workers from the internal source to the external sink. This unidirectional pull may be an important factor in the structure and organisation of division of labor (Tofts and Franks 1992; Tofts 1993). While external workers may themselves be divided into different task groups, in most species the final group and thus the main sink are the foragers (Porter and Jorgensen 1981; Gordon 1986). Consequently, any change in the mortality rate of external workers may lead to a relatively simple change in the linear flow of workers from internal to external tasks.

Systems with more complex flow patterns between sources and sinks are likely to provide us with novel insights into the structuring and organization of division of labor. Leaf-cutting ants with an external waste heap (e.g. Atta colombica (Weber 1972) where waste is composed mainly of discarded fungus garden material) embody just such a complex system, with two distinct and well-characterised external sinks – foragers and waste workers (Hart and Ratnieks 2002). At any one time, foragers represent 88.8% of external workers, with waste workers making up the remainder (Hart and Ratnieks 2002). It is likely that waste heap management increases nest hygiene and reduces disease transmission within the colony, and that a failure to allocate workers to waste management tasks would be highly detrimental to the colony (Hart and Ratnieks 2001, 2002). Waste workers are further sub-divided into waste transporters, carrying waste from inside the nest to the external waste heap, and waste heap workers (0.2% of the total external work force), which remain on the heap and work with the waste, presumably speeding its decay. Transitions between foraging and waste heap working are very rare and the reverse transition has not been observed (Hart and Ratnieks 2002). Thus, internal workers may follow one of two routes to an external task – either to become a forager or to become, first, a waste transport worker and then a heap worker. Consequently, the dynamics of worker flow from internal to external tasks should depend, to some extent, upon mortality rates in the two spatially-separated and physically-differentiated tasks of foraging and waste-working. While from a static numerical perspective foraging would appear to be the most important sink in this system, a relatively high rate of mortality in waste workers could have a significant impact on the flow of workers between internal and external task groups.

Mortality rates in external workers have two components. First, the intrinsic rate of mortality due, for example, to worker age. Second, additional mortality imposed by the task-related environment, for example, the energy cost of doing a task or exposure to predators. In this study, we take the first steps in addressing how mortality rates across task groups may affect division of labor. Taking A. colombica as our model system, we asked a) do intrinsic mortality rates vary across external task groups?, and b) does exposure to a task-related environment result in increased mortality rates among waste-heap workers? The results are discussed in relation to field observations of division of labor in this species, and more generally in the context of the evolution of division of labor in social insects.

## Methods

Experiments were carried out in April/May 2000 at the Smithsonian Tropical Research Institute field station, Gamboa, Panama. Ants for the experiments were collected from mature colonies of Atta colombica on the day that the experiment was set up. We conducted two, separate experiments.

### Experiment one – intrinsic mortality rates across task groups

To determine the intrinsic mortality rates of ants involved in different tasks, we carefully collected, using soft forceps, a total of 449 ants from each of three different task groups from four different colonies (for task-group definitions, see below) (Colony 1 – Foragers = 50, Transporters = 49, Heap workers = 46; Colony 2 – Foragers = 41, Transporters = 41, Heap workers = 21; Colony 3 – Foragers = 39, Transporters = 40, Heap workers = 20; Colony 4 – Foragers = 40, Transporters = 40, Heap workers = 22). Sample sizes for Heap workers were limited by the number of ants engaged in this task. We allocated ants to task groups by reference to where they were collected and what they were doing (Foragers – collected on foraging trail returning with leaf fragments; Transporters – collected travelling along trails from the colony to the waste pile and carrying waste; Heap workers – collected on the waste heap where they were manipulating waste). Ants were placed individually into ventilated glass jars and kept in the laboratory under 100% relative humidity, 30°C and darkness. Ants had no access to food, but the humidity gave them *ad libitum* access to water. In the natural environment, heap workers have no access to the food sources needed to enhance survival (leaves and fungal garden material, Silva et al. 2003), transport workers are similarly constrained (although it is possible that they may be fed by garden workers), while foragers at least have access to leaves. Consequently, our methods underestimate survival (and thus overestimate mortality rates) of foragers and, possibly, transporters. Mortality checks were conducted every three hours until all animals were dead, and at additional haphazard times between checks. The mass of dead ants was measured to the nearest 0.1 mg. We used a Cox regression survival analysis to investigate mortality during the experiment. Survival time was the dependent variable, and task, colony, ant body mass, and all their two- and three-way interaction terms were presented as candidate variables. Colony and task were coded as deviation variables (as there was no obvious control or baseline treatment), with weight as a continuous variable.

### Experiment two – intrinsic vs. task-related environment induced mortality in heap workers

We collected a total of 120 ants from each of three new colonies. We collected only Transporters, as this group of workers eventually produces the Heap workers and thus our experiment simulates the arrival of a worker in the heap worker task group. Using soft forceps, we collected the first 120 ants to cross an imaginary line drawn across the transport trail. We also collected waste from the waste heap of each colony, making sure to collect both old and newly–deposited waste (the two types are easily distinguished: old waste is darker than fresh waste and is located more peripherally on the heap). Heap workers spend time on both types of waste (personal observations) so, by collecting both types we could better simulate the environment experienced by Heap workers.

We set up 3 laboratory treatments for each colony. In the Control treatment, we placed 10 ants in a covered petri dish (9 cm diameter) without waste (3 replicates per colony). In the Own treatment, we placed 10 ants in a petri dish, after covering the bottom of the dish with a mixture of old and new waste from their own colony (again, 3 replicates). In the Foreign treatment, we placed 10 ants in a petri dish, after covering the bottom of the dish with a mixture of old and new waste from one of the other colonies (3 replicates x 2 colonies). This treatment was included to determine whether any effects of exposure to waste on mortality were colony-specific. To summarise, we set up a total of 36 petri dishes (3 colonies X 4 treatments [Control + Own + 2 X Foreign] X 3 replicates) containing a total of 360 ants. The control treatment enabled us to determine mortality rates for ants in the absence of task-related environment, while the Own and Foreign waste treatments provided data on mortality rate when workers were exposed to such an environment. Any additional mortality due to the environment could be caused directly by toxic/pathogenic effects of exposure to waste, or due to an increased metabolic rate caused by the performance of task-related behaviour (e.g., waste sorting). Either way, our experiment provides a general measure of waste-related mortality. After set-up, all petri dishes were kept under constant conditions of 100% relative humidity, 30˚C and darkness. As with experiment 1 (see above), ants were starved but had *ad libitum* access to water. Dishes were checked for ant mortality every six hours until all ants were dead. When dead ants were found, they were removed from the petri dish (to prevent contamination) and weighed to the nearest 0.1 mg.

Mortality data were analysed using a Cox regression survival analysis. Survival time was the dependent variable, and treatment, colony, ant body mass, and all their two- and three-way interaction terms were presented as candidate variables. Treatment and colony were coded using indicator and deviation coding, respectively, with the control treatment as the reference category. Ant body mass was a continuous variable.

All statistical analyses were done using SPSS 10 for the Macintosh.

## Results

### Experiment one – intrinsic mortality rates across task groups

**Figure 1:**
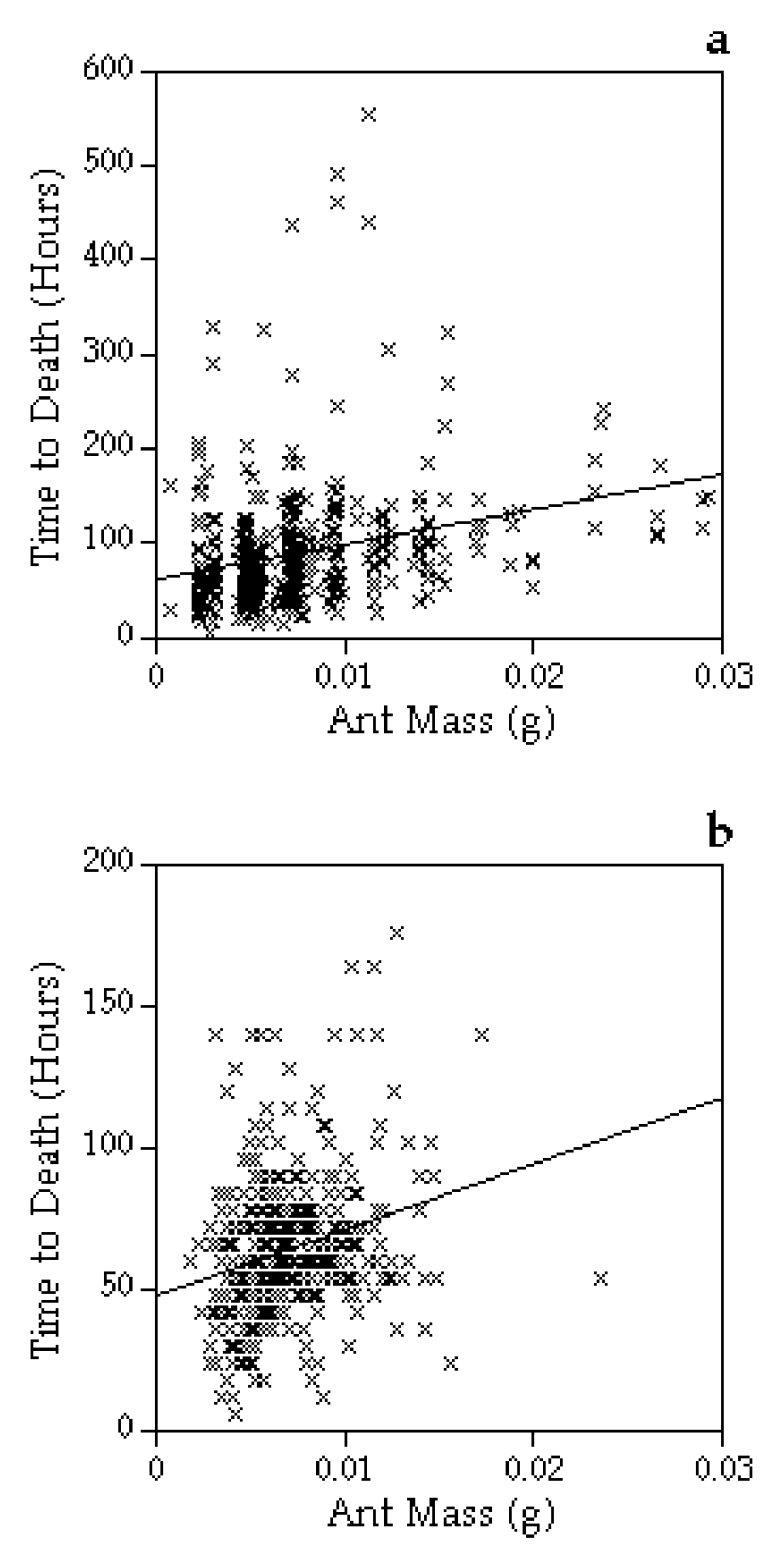
The linear relationship between ant (Atta colombica) mass and mortality in experiments one (a) and two (b). In both experiments, as ant mass (*x*-axis) increased the time to mortality (*y*-axis) also increased. The lines shown are best fit lines (a: *y* = 3744*x* + 61; b: *y* = 2336*x* + 47) and do not reflect the exact survival function attributed to ant mass by the Cox regression survival analysis. Each cross represents a single ant. See text for statistical analyses.

The mortality rate of workers did not depend directly upon the task-group from which they were collected. There was no effect of task-group in the final Cox regression survival model (DF = 2, Likelihood score = 2.471, *P* = 0.291). Similarly, there was no direct effect of a worker’s colony of origin on mortality (DF = 3, Likelihood score = 5.054, *P* = 0.168). In contrast, body size, as measured by weight, had a significant effect, with larger workers living longer (Fig 1a; DF = 1, Wald statistic = 36.9, *P* < 0.001).

While task-group and colony of origin had no direct effects on mortality, there was a significant interaction between these factors (DF = 6, Wald statistic = 27.6, *P* < 0.001). This was mainly due to Foragers from colonies 1 and 2 having, respectively, 127% higher (DF = 1, Wald statistic = 11.2, *P* = 0.001) and 41% lower (DF = 1, Wald statistic = 5.1, *P* = 0.024) mortality rates than the average. There was also a significant 3-way interaction among task-group, colony of origin and worker size (DF = 6, Wald statistic = 16.4, *P* = 0.012), mainly due to increased survival in larger Foragers from colony 1 (DF = 1, Wald statistic = 4.3, *P* = 0.038) and larger Transporters from colony 3 (DF = 1, Wald statistic = 5.6, *P* = 0.018).

### Experiment two – intrinsic vs. task-related environment induced mortality in heap workers

Observations of workers and, in the Own and Foreign treatments, waste during mortality checks showed that workers were sorting waste and thus conducting task activities when given the opportunity. In the control treatment, workers appeared generally inactive.

**Figure 2:**
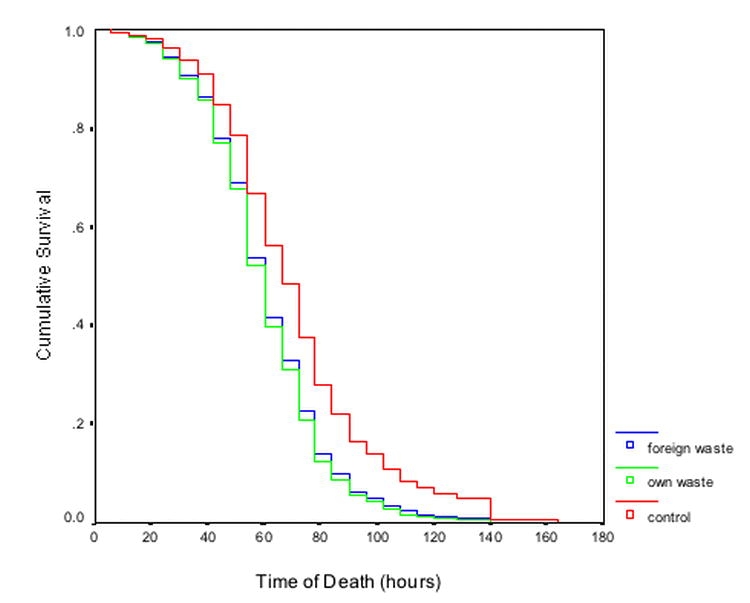
Mortality rate varies across the three waste material treatments. The rate of mortality for control ants (Atta colombica) (without waste material; red line to the right) was slower than for the Own (green line) and Foreign (blue line) waste treatments (these two lines overlap on the graph). The x-axis shows time in hours and the y-axis shows survival curves for the three treatments. Curves represent the survival functions calculated by the Cox regression survival analysis. See text for statistical analyses.

Exposure to waste significantly increased the mortality rate of workers (Fig 2; DF = 2, Wald statistic = 11.6, *P* = 0.003). This was true both for workers with waste from their own colony (58% increase over controlled workers; DF = 1, Wald statistic = 8.4, *P* = 0.004) and workers with waste from foreign colonies (52% increase; DF = 1, Wald statistic = 9.3, *P* = 0.002). There was no difference in the mortality rate of the two waste treatments. As with experiment one, there was also a significant effect of body size, with heavier workers living longer (Fig 1b; DF = 1, Wald statistic = 30.0, *P* < 0.001), but no effect of colony-of-origin (DF = 2, Likelihood score = 2.8, *P* = 0.253).

There was a significant interaction between colony of origin and waste treatment (DF = 4, Wald statistic = 28.5, *P* < 0.001), mainly due to ants from colony 1 exposed to foreign waste having a relatively lower mortality rate (DF = 1, Wald statistic = 18.4, *P* < 0.001). There was also a significant 3-way interaction among waste treatment, colony of origin and ant size (DF = 4, Wald statistic = 22.39, *P* < 0.001), mainly due to increased survival in larger workers from colony 1 exposed to foreign waste (DF = 1, Wald statistic = 15.8, *P* < 0.001).

There was no significant difference across the two experiments in either the mortality rate of Transporters (using only the Control individuals from the second experiment; the final logistic regression model contained ant mass as the only significant predictor of mortality rate) or the mean time to death (*F_1,257_* = 1.396, *P* = 0.239), indicating that isolation of individual ants in experiment one did not affect their mortality.

## Discussion

Working on the waste heap of a leaf-cutting ant colony is clearly a costly business. Worker mortality rates were nearly 60% higher when ants were exposed to, and allowed to work, waste material than when left in control conditions.

In this study two sources of mortality were analysed in external workers. First, intrinsic mortality rate did not differ across task groups. If this were the only source of mortality, it would indicate that foraging is by far the largest sink for a colony’s workers. In these experiments, ants had no access to food, but work by Silva et al. (2003) shows that foragers of Atta sexdens, a closely related species to A. colombica, are likely gaining nutrition from either leaves, fungal garden material, or both, which acts to prolong their lifespan. Consequently, for foragers our experimental protocol almost certainly underestimated potential lifespan. While it is unclear whether waste transport workers have any source of nutrition (their activity leads to them being isolated from the rest of a colony’s workers, to prevent contamination of the fungal garden), waste heap workers have no access to either source of nutrition. These considerations suggest that mortality in waste heap workers may be more important as a sink for a colony’s workers than our results suggest (see below for further discussion).

In the second experiment, the first step was taken towards understanding task-specific additional mortality, which showed that waste-heap workers have a 60% increased mortality rate when exposed to their task-related environment. This increase in mortality either results from an increase in metabolic activity due to sorting and working waste, or from exposure to pathogenic organisms present in waste (including mites and fungi, Bot et al. 2001), or, most likely, a combination of both factors. It would be interesting to conduct further experiments using sterilised waste material to separate these two sources of task-related mortality. In addition, a complete description of this system would require further laboratory and field studies to measure task-related mortality rates in foragers and waste transporters.

What are the implications of these results for understanding division of labor? This work, together with Hart and Ratnieks (2002), provides the first demonstration of a division of labor system with two costly and thus potentially important worker sinks. In Atta colombica, foragers and transport/heap workers do not belong to distinct morphological castes (Hart and Ratnieks 2002) and thus presumably draw on a common pool of reserves within the nest. Consequently, decisions about allocating workers to foraging must be traded off against allocating workers to waste management work. This contrasts with previously studied systems, where only one sink exists and transition of workers occurs from midden work (equivalent to heap work in this study) to foraging work (Porter and Jorgensen 1981; Gordon 1989). While we have shown that such a trade-off exists in our study system, how important is it? A simple acceptance of the mortality data from experiment 1 would suggest that mortality due to heap work is unlikely to play a major role in regulating this trade-off, given the small number of workers involved in this task. However, if we have overestimated mortality in foragers, as the work of Silva et al. (2003) would strongly suggest, it is possible that the increased rate of mortality in active heap-workers may indeed play an important role in the flow of workers between tasks. Further work, on both this and the importance of waste transport work vs. foraging, is needed.

The mortality rates suffered during heap work also affect the movement of workers between waste transport and waste heap work. The population of waste transporters is 55 times larger than that of heap workers (Hart and Ratnieks 2002). This suggests that either waste transporters have remarkably low task-related mortality rates or, more likely, many workers die before having the opportunity to become heap workers. If the latter is true, then waste transport, not heap work, would be the main second sink for workers and assessment of mortality rates in waste transporters and foragers is essential to really understand the dynamics of this two sink system.

However, it is under conditions of colony-stress that waste-related mortality is likely to have the biggest impact. Hart et al. (2002) showed that large numbers of heap workers were only present when the danger posed to a colony by the dangerous parasitic fungus Escovopsis was high (we note that this also supports the idea that the costs of heap work are sufficient for colonies to modulate task allocation with respect to them). In such conditions, where large number of heap workers are needed, there may yet be effects for the whole system, with heap workers pulling more workers through from the transport group, and potentially reducing the number of workers available for foraging.

We also found a significant effect of worker size on mortality rates, with larger ants living longer. It seems likely that this effect was due to larger ants having greater energy stores and thus being able to resist mortality for longer. Such lifespan/size patterns have been found under other circumstances (Porter and Tschinkel 1985), leading to the suggestion that larger ants are more valuable to a colony as they can do a task for longer than their smaller sister workers (Hölldobler and Wilson 1990).

So far, we have implied that the impact of worker sinks on division of labor and task allocation is a simple relationship, with smaller sinks drawing fewer workers through and vice versa. However, in reality task allocation and division of labor are complex dynamic systems, which require both positive and negative feedback loops in order to function. It seems likely to us that if mortality becomes too high, tasks that previously acted as sinks may then inhibit the movement of workers from one task group to another, leading to the shutting down of colony functions (Whitford and Bryant 1979; MacKay 1982; Greene and Gordon 2003) and potentially the initiation of new activities, such as nest migration away from the cause of mortality (Hart 2002).
